# Peptidyl transferase center decompaction and structural constraints during early protein elongation on the ribosome

**DOI:** 10.1038/s41598-021-02985-7

**Published:** 2021-12-15

**Authors:** Bin Jia, Tianlong Wang, Jean Lehmann

**Affiliations:** 1 Department of Anesthesiology, Xuanwu Hospital, Capital Medical University, Beijing, China; 2 CEA, CNRS, Institute for Integrative Biology of the Cell (I2BC), University of Paris-Saclay, 91198 Gif-sur-Yvette, France

**Keywords:** Translation, Ribosome

## Abstract

Peptide bond formation on the ribosome requires that aminoacyl-tRNAs and peptidyl-tRNAs are properly positioned on the A site and the P site of the peptidyl transferase center (PTC) so that nucleophilic attack can occur. Here we analyse some constraints associated with the induced-fit mechanism of the PTC, that promotes this positioning through a compaction around the aminoacyl ester orchestrated by U2506. The physical basis of PTC decompaction, that allows the elongated peptidyl-tRNA to free itself from that state and move to the P site of the PTC, is still unclear. From thermodynamics considerations and an analysis of published ribosome structures, the present work highlights the rational of this mechanism, in which the free-energy released by the new peptide bond is used to kick U2506 away from the reaction center. Furthermore, we show the evidence that decompaction is impaired when the nascent peptide is not yet anchored inside the exit tunnel, which may contribute to explain why the first rounds of elongation are inefficient, an issue that has attracted much interest for about two decades. Results in this field are examined in the light of the present analysis and a physico-chemical correlation in the genetic code, which suggest that elementary constraints associated with the size of the side-chain of the amino acids penalize early elongation events.

## Introduction

During protein synthesis on the ribosome, the peptidyl-transferase center (PTC) sequentially catalyzes the formation of new peptide bonds between incoming aminoacyl tRNAs and a growing peptide passed on from tRNA to tRNA. Unlike most enzymes, the ribosome has to deal with a large variety of substrates in the form of aminoacyl-tRNA, where the L side-chain of the aminoacyls can be absent (glycine) or very bulky (e.g. tryptophane), and exhibit extreme properties in terms of charge distribution and hydrophobicity. It implies that no direct interaction between the PTC and the side chain could occur to orient an aminoacyl for nucleophilic attack. Instead, the PTC achieves this positioning through an induced-fit mechanism^1^ that creates a compaction around the C_α_ of the aminoacyl ester, freezing it in an appropriate orientation almost without interfering with the side chain^2^. As the compact state has only recently been recognized as such, the issue of PTC decompaction after peptide bond formation, that allows the A-site peptidyl tRNA product to get free and move to the P site, has not been investigated so far.

Based on a survey and examination of ribosome structures complexed with various arrest peptidyl-tRNA and peptidyl-tRNA analogs retrieved from the pdb database, and from free-energy estimates, the present work shows that some of the free-energy released by the reaction of peptide bond formation is used to decompact the PTC, which allows the peptidyl-tRNA to freely move to the P site. Furthermore, we point out that structural constraints associated with decompaction when the nascent peptide is short may contribute explain why protein synthesis is impaired during the first rounds of elongation. A reanalysis of expression data with eGFP reporters published by Djuranovic et al.^3^ was motivated by a correlation between the AU content of codons 3–5 and eGFP scores established by these authors. Because weak (i.e. AU-rich) anticodon-codon interactions encode large amino acids^4^, we investigated the possibility that the size of the side-chains may condition the outcome of these early elongation events. Our analysis shows that very large and very small amino acids are inhibiting in that respect, suggesting that basic physical constraints are associated with the growth of tiny nascent peptides. Furthermore, a set of data published by Gelfand and Sergiev et al.^5^, centered on positions 2 to 11, show that the various effects of the amino acids on translational efficiency become essentially uniform starting from position 7, suggesting that nascent proteins are anchored inside the exit tunnel from that size.

## Results

### A compaction–decompaction cycle in the PTC

**Figure 1:**
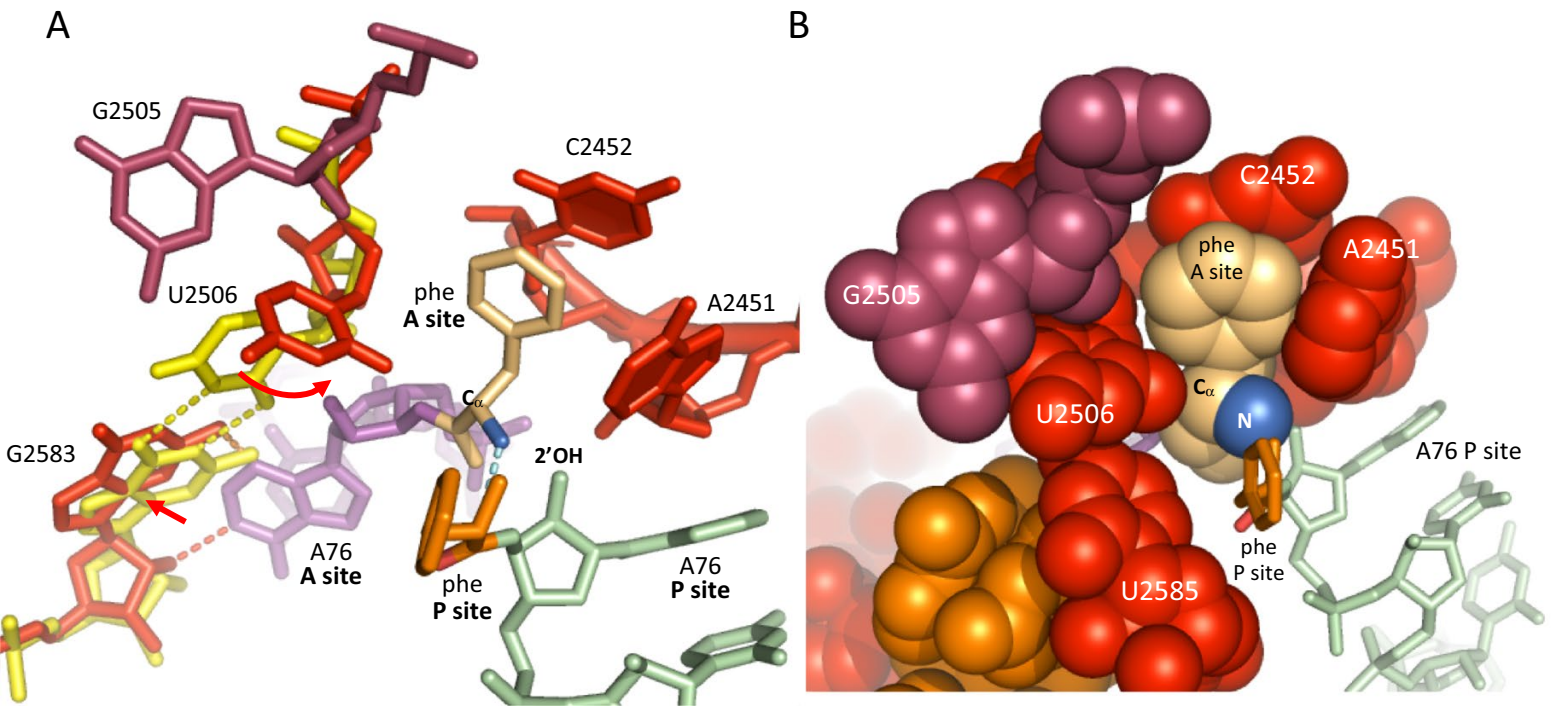
Compaction of the PTC around the incoming aminoacyl ester. (**A**) Induced-fit mechanism of the PTC^1^. In the absence of A-site substrate, U2506 preferentially* wobble base pairs with G2583 (yellow dotted lines) (pdb 5kcr)^11^. This interaction brakes when the terminal A76 of an incoming tRNA binds to and shifts the position of G2583 (orange dotted lines and red arrow). As a result, U2506 moves to another equilibrium position (curved red arrow) that squeezes the Cα carbon against the 2ʹOH of the A76 of the P site tRNA (pdb 4v5d)^12^, settling the amino group into a proper orientation for nucleophilic attack (blue dotted line). Superimposition achieved with *Pymol*. (**B**) Situation after induction, with atoms shown with van der Waals radius. The PTC wraps around the aminoacyl ester (pdb 4v5d)^12^. *An examination of ribosome structures reveals that U2506 does not always base pair with G2583 in the absence of A-site tRNA (Y. Polikanov, pers. comm.). However, in the presence of both A-site and P-site tRNAs substrates, U2506 is always found in an induced position which contains the Cα carbon of the amino acid. The PTC superimposition shown in A involves *E. coli* (pdb 5kcr) and *T. thermophilus* (pdb 4v5d) ribosomes. The PTC being highly conserved, all residues involved in the induced-fit mechanism are identical in both species.

The induced fit of the PTC^1^ is the mechanism by which ribosomes handle the flexibility of the ester bond linking the amino acid to the tRNA, a property that studies on intramolecular reactions have shown to drastically impair nucleophillic attack^6–9^. Upon binding of the CCA 3ʹ end of an incoming tRNA on the A site, the G2583-U2506 wobble base pair that usually keeps U2506 away from the reaction center breaks, forcing U2506 to adopt a conformation jamming the C_α_ of the aminoacyl ester against the A76 2ʹOH of the P site tRNA (Fig. 1A). This conformational change (or, perhaps more accurately, conformational selection), that orients the amino group for nucleophilic attack, generates a compaction around the amino acyl almost without interfering with the side chain (Fig. 1B). As a result of this compaction, amino acids with large side chains, such as phenylalanine or tryptophan, are literally trapped inside the PTC. This structural rearrangement suggests that the thermodynamic cost for orienting the nucleophile of the aminoacyls is ultimately provided by the binding of the CCA of the A-site tRNA at the entrance of the PTC. Furthermore, the confinement achieved by the PTC results in a downshift the *pk*_*a*_s of the amino groups due to desolvation^10^, thus making them more reactive at physiological pH.

**Figure 2:**
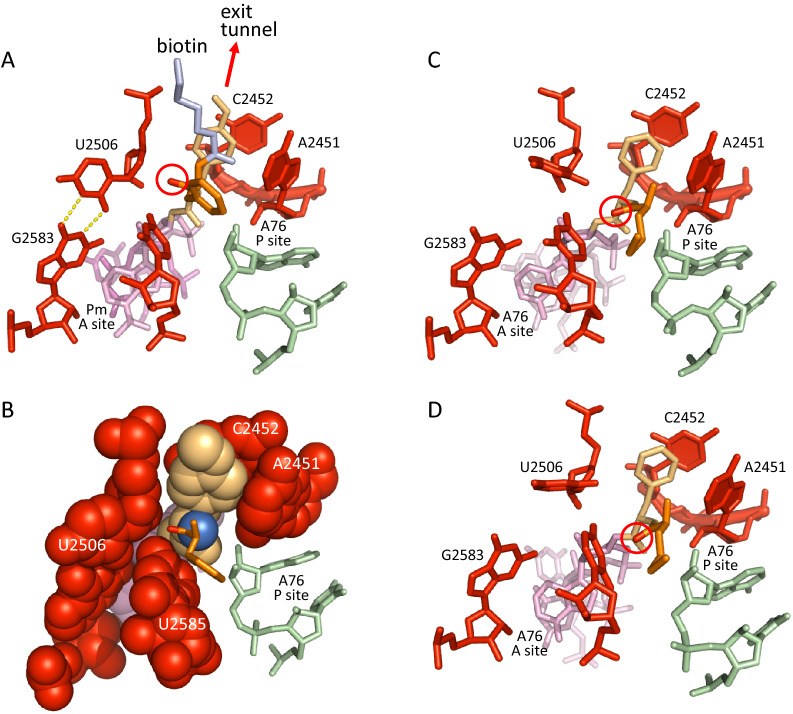
Decompaction of the PTC upon peptide bond formation. (**A**) Crystal structure of a CC-puromycin-dipeptide anchored in the cavity of the PTC by a biotin (pdb 1kqs)^13^, representing the situation just after peptide bond formation, when the 3ʹ end of the A site tRNA has not yet moved to the P site (res.: 3.1 Å). The biotin linker (shown in blue grey) keeps the dipeptide in a stretched configuration oriented towards the exit tunnel (red arrow). (**B**) Same structure as in A, with van der Waal representation (the biotin linker is not shown). (**C**) Crystal structure of a dipeptidyl-tRNA bound to the A site of the ribosome (pdb 1vy5) (res.: 2.55 Å)^18^. (**D**) Cryo-EM structure of a dipeptidyl-tRNA bound to the A site of the ribosome (pdb 6wde) (res.: 3.0 Å)^19^. The carbonyl oxygens of the peptide bonds are circled in (**A,C,D**).

Having achieved substrate positioning through compaction, how could the PTC get out of that state (Fig. 1B)? The pdb 1kqs structure^13^, in which a CC-puromycin-dipeptide connected to a biotin is bound on the A site, suggests how decompaction occurs. This structure corresponds to a situation in which the A-site CCA-peptide that results from peptide bond formation has not yet move to the P site. It reveals that U2506 is kicked out of the induced state by the carbonyl oxygen of the new peptide bond (Fig. 2A,B), suggesting that the free-energy required to decompact the PTC comes from the chemical reaction itself, an aspect that was not investigated in the original study^13^. Thermodynamic data on tRNA aminoacylation fully concur with this possibility. In this two-step reaction, mediated by the aminoacyl tRNA synthetases, an amino acid and an ATP are first converted into aminoacyl adenylate (aa-AMP), an activated form which can react and deliver the amino acid to the 3ʹ end (or 2ʹ end) of a tRNA. The equilibrium constant *K*_*eq*_ of the whole process was found to be ~ 0.3 to 0.7 (as determined with resp. valine and threonine), indicating that the free-energy available in the ester bond connecting an amino acid to the 3ʹ end of a tRNA is about equivalent to that of a phosphodiester bond of ATP^14^. The free-energy of ATP to ADP + pi hydrolysis is about − 7.5 kcal mol^−1^ under standard conditions, while the hydrolysis of a peptide bond is − 2 to − 4 kcal mol^−1^^15^. These two sets of values allow to estimate the free-energy change of peptide bond formation, which is − 7.5 to (− 2 to − 4) =  − 5.5 to − 3.5 kcal mol^−1^ (a value of − 3·7 ± 1·2 kcal mol^−1^ was reported in Noller et al.^16^).

Part of this free-energy is available to perform some work required to drive the process forward: PTC decompaction and tRNA CCA 3ʹ end translocation. While the possibility that the free-energy associated with 3ʹ end translocation may come from peptide bond formation has already been extensively discussed^16,17^, the issue of PTC decompaction has not been considered so far. Our analysis suggests that a fraction of the free-energy released by peptide bond formation is used on the ribosome to decompact the PTC through a kind of power stroke, in which the carbonyl oxygen of the new peptide bond kicks U2506 away from the reaction center (Fig. 2A,B). A conclusion from the above estimates is that the free-energy stored in the ester bond connecting the aminoacyl to a tRNA is essential to getting product release through PTC decompaction, implying that the amount of free-energy left to drive CCA 3ʹ end translocation is necessarily reduced. The translocation of the CCA 3ʹ end, however, does not require additional free energy, while the translocation of the anticodon-codon complex, which is trapped by the decoding center, requires the action of EF-G and the hydrolysis of a GTP to ensure directionality^16^. Protruding obstacles on the path from A site to P site, such as residue A2602, may also explain why input(s) of free-energy are required for tRNA translocation.

Remarkably, the 1kqs structure shows that the initial G2583-U2506 wobble base pair is restored upon decompaction, even though the CC-puromycin triplet is still bound to the A loop, and thus keeps the G2583 residue in the displaced configuration. The above analysis suggests that this unfavourable configuration may help release the CCA triplet from the bound state, and thus facilitate the translocation of the peptidyl-tRNA to the P site of the PTC, a process which is spontaneous^20^.

### Ribosome structures suggest that decompaction is impaired during the early rounds of elongation

**Figure 3:**
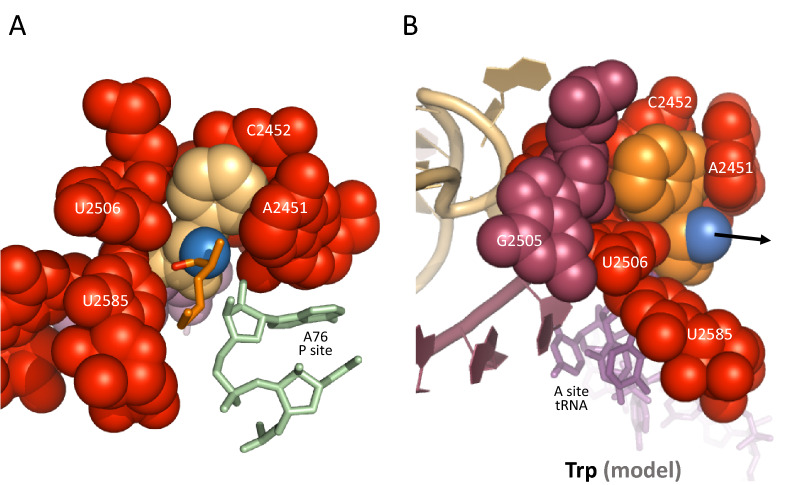
Constraints on translocation with partially decompacted PTC. (**A**) This view reveals that partial decompaction of the PTC may interfere with the translocation of the 3ʹ end from the A site to the P site (most atoms are shown as van der Waals spheres) (pdb 1vy5)^18^. To compare with Fig. 2B. (**B**) Induced PTC with tryptophanyl-tRNA on the A site of the ribosome. This configuration was obtained with a Phe → Trp mutation in the pdb 4V5D structure^12^ as the only modification. It suggests that very large amino acids such as tryptophan are trapped in induced (compacted) PTC, which may prevent CCA 3’ end translocation (arrow) if peptide bond formation would not promote decompaction.

**Figure 4:**
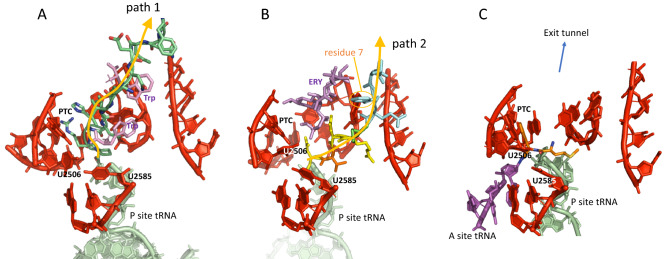
Arrest peptides and dipeptidyl-tRNA the ribosome. (**A**) Trp-arrest peptide following path 1, with peptidyl-tRNA on the P site and two bound tryptophan (Trp) (pdb 4UY8^21^). (**B**) Erythromycin-arrest peptide following path 2, with peptidyl-tRNA on the P site and a bound erythromycin (ERY) (pdb 5JU8^11^). Amino acid No. 7 (Phe, highlighted) is at the level of U2609 (in the foreground, not shown). (**C**) Dipeptidyl-tRNA on the A site of the ribosome (pdb 1VY5^18^). Path 1 and 2 are discussed in Arenz et al.^11^.

To get further insights into the mechanism of decompaction, and highlight possible effect(s) associated with tiny nascent peptides, we have examined structures similar to 1kqs, but where the A-site tRNA only carries a dipeptide (instead of a polypeptide already anchored in the exit tunnel). We found that decompaction is impaired in that case, as revealed by structures 1vy5 and 6wde (Fig. 2C,D). Both cryo-EM and X-ray structures show that U2506 is only slightly displaced from the induced state, the reason being that the dipeptide is in a more relaxed configuration, and leans towards the P site. As a result, the orientation of the carbonyl oxygen of the new peptide bond is tilted, and cannot optimally kick U2506 to make it pass over U2585, this residue being involved in keeping U2506 in the induced state (Fig. 1B). In comparison, the 1kqs structure shows that the carbonyl oxygen of the new peptide bond is ultimately the reason why U2506 is kicked out of the induced state. Although this structure does not include an actual peptidyl-tRNA, the orientation of the dipeptide towards the exit tunnel enforced by the biotin, that mimics the situation with a true elongated peptide, highlights the mechanical effect of such constraint on the dipeptide. As far as we are aware of, there is no cryo-EM or X-ray ribosome structure with a peptide longer than a dipeptide with the CCA still bound to the A site, which could confirm the situation found with the 1kqs structure. In the same way as for the 1vy5 structure, such configuration could be obtained by letting a synthetic peptidyl-tRNA and aminoacyl-tRNA react on the ribosome, and get X-ray or cryo-EM data from the resulting complex. The above observations, however, suggest that incomplete decompaction might impair the translocation of the A-site tRNA CCA-peptide to the P site during the early rounds of elongation, when the peptide is not yet anchored in the exit tunnel (Fig. 3). Anchoring would keep it in a stretched configuration, similar to the situation found with the 1kqs structure, and thus ensure an efficient decompaction of the PTC upon peptide bond formation. Furthermore, with U2506 not pairing with G2583, the terminal CCA-peptide could less easily detach from the A loop (see above). Ribosome structures with arrest peptides have revealed that different paths can be taken by nascent peptides^11,21^. At least two paths have been identified (Fig. 4): a direct path (path 1), where the peptide follows a rather straight trajectory, and a trajectory (path 2) which is followed when a macrolide (erythromycin) blocks the direct path^11^. Interestingly, although path 2 has been established with an arrest peptide, it was shown to be viable in *S. aureus* with ribosomes-bound erythromycin in which mutations in protein uL22 widens the tunnel away from the PTC^22^. These data show that different paths may be taken by nascent peptides, and one would thus expect that some peptides may not adopt a proper trajectory that would allow them to get easily inserted into the exit tunnel, which may slow down or prevent a proper rearrangement of the CCA-peptide on the P site, required for subsequent peptide bond formation.

### Inefficient decompaction and nascent peptide conformational freedom may explain the translational ramp

Early elongation events (called the translational ramp) were found to be critical to the yield of protein synthesis, an issue that has attracted much interest for about two decades. Studies have highlighted that the codons/amino acids immediately following position 1 determine to a large extent the total amount of protein synthesized^5,23–26^. In an exhaustive investigation in which several biochemical aspects were considered, Verma et al.^3^ highlighted the criticality of codons 3 to 5 and their corresponding amino acids using an *E. coli* expression system, where the effect on protein synthesis was established with an eGFP reporter. In brief, single inserts encoding a eGFP peptide preceded by an initiation sequence with pos 3, 4 and 5 randomized were expressed in bacteria, where it generated a certain level of fluorescence depending on the amount of peptide produced. These bacteria were sorted into 5 different bins according to the level of fluorescence (quantified hereafter under the term “eGFP scores”) and subsequently sequenced to determine the identity of the codons/amino acids in pos 3–5^3^.

**Figure 5:**
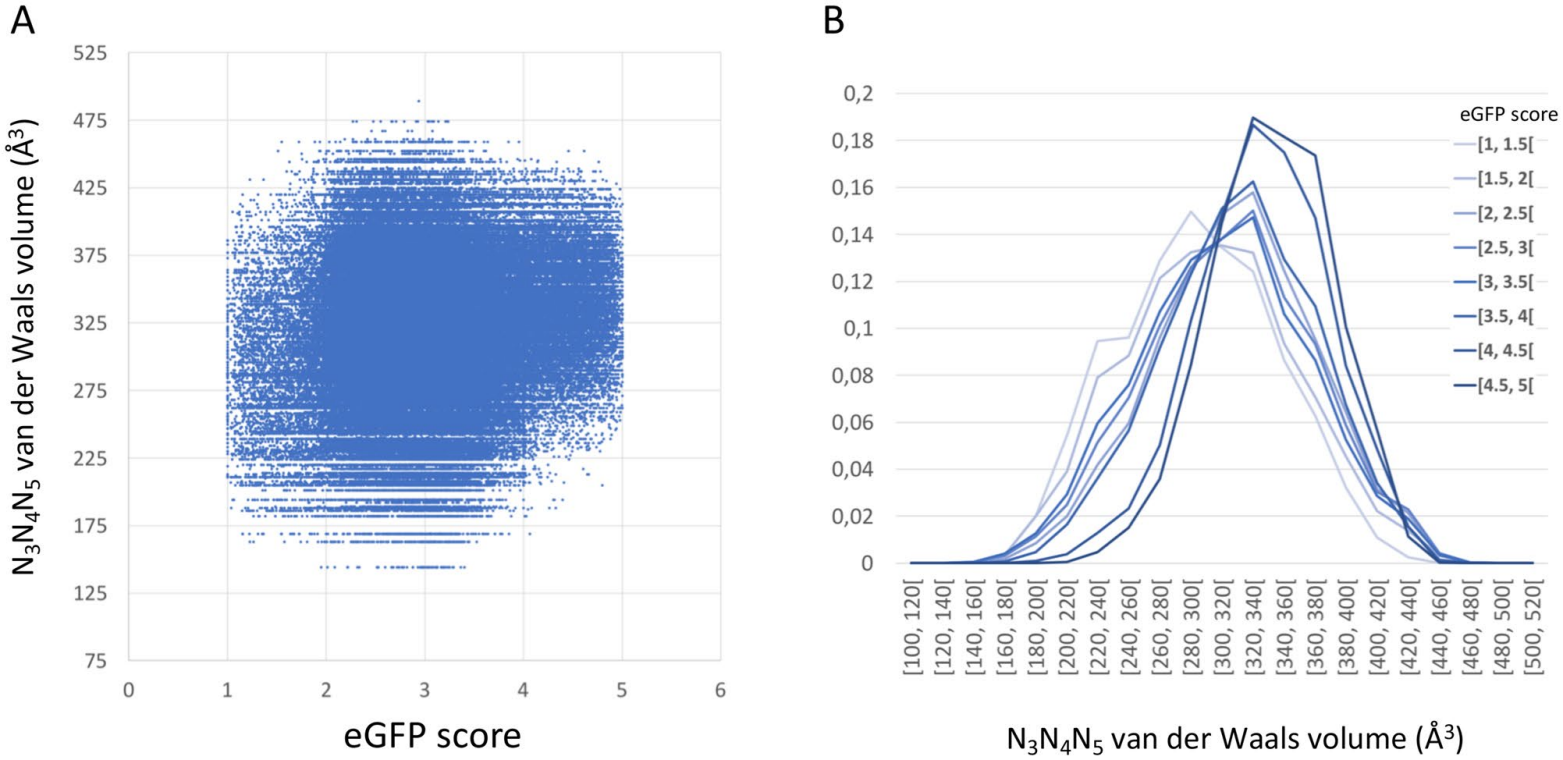
Van der Waals volume of N_3_–N_5_ triplets as a function of the expression of mRNAs bearing these triplets, established from the fluorescence of eGFP reporters expressed in *E. coli* cells. Plots established from the values listed in Supplementary Data 3 of the work published by Djuranovic et al.^3^. Van der Waals volume values are from Ref. ^60^. (**A**) N_3_–N_5_ van der Waals volume as a function of all eGFP scores. (**B**) Normalized distributions of eGFP scores as a function of N_3_–N_5_ van der Waals volumes, established for different eGFP intervals. See Ref.^3^ for details about experimental procedures.

**Figure 6:**
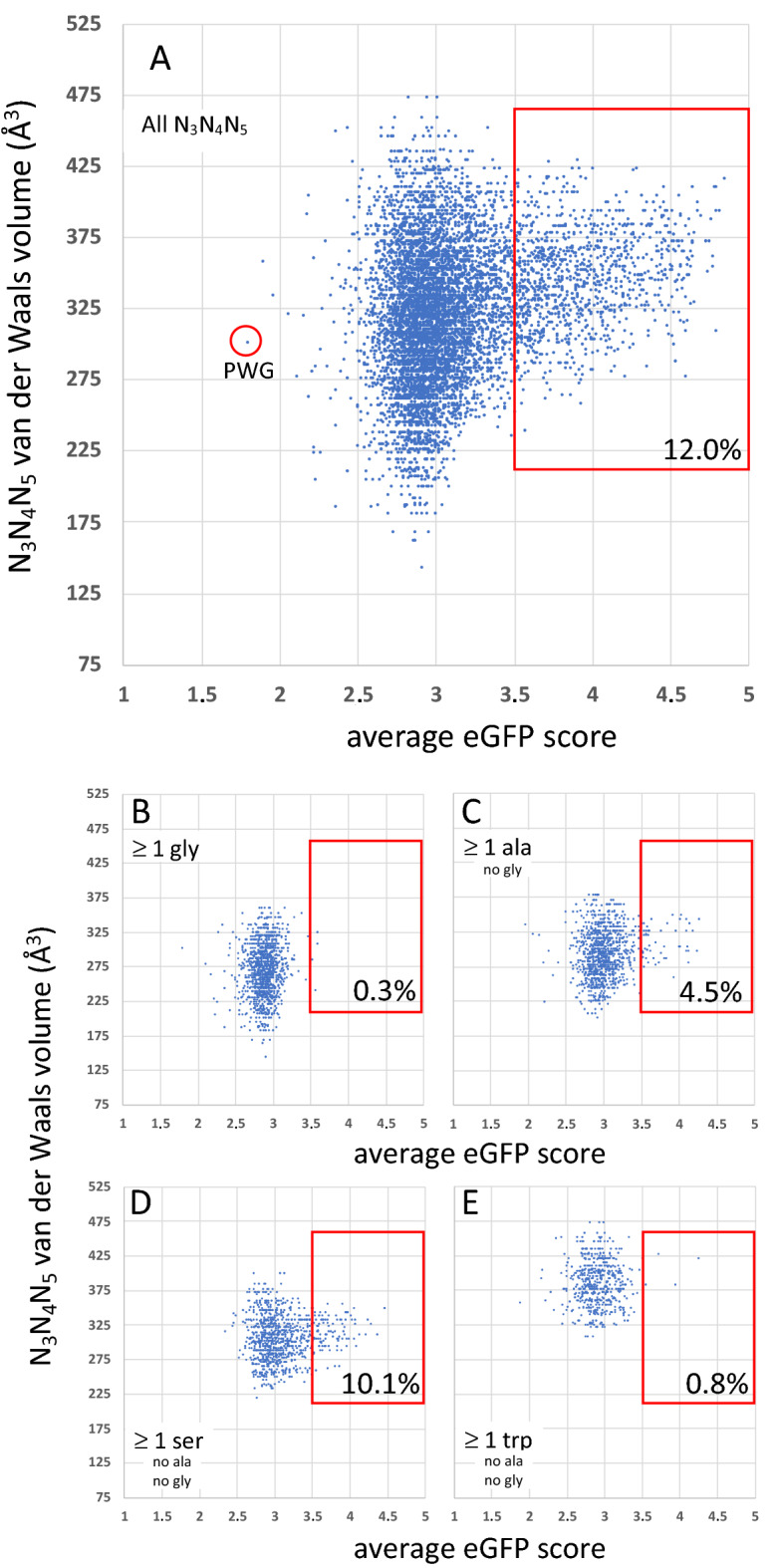
Van der Waals volume of N_3_–N_5_ triplets as a function of average eGFP scores (see Fig. 5). Only triplets for which at least 5 independent eGFP scores were reported are included (Supplementary Data 3 from Ref.^3^). (**A)** Plot with all N_3_–N_5_ triplets. (**B**) Plot for all triplets that include at least one glycine. (**C**) Plot for all triplets that include at least one alanine and no glycine. (**D**) Plot for all triplets that include at least one serine, without any glycine and alanine. (**E**) Plot for all triplets that include at least one tryptophan without any glycine and alanine. The fraction of eGFP scores equal or higher than 3.5 are highlighted on the plots.

Here we discuss some of their results in the light of our observations on PTC decompaction and a physicochemical correlation in the genetic code. These authors pointed out that the percentage of (A + U) nucleotides within codons 3 to 5 correlates with protein expression^3^. Because AU-rich codons encode large amino acids^4^, we predicted that the corresponding tripeptides in pos. 3–5 would display voluminous side-chains compared to those encoded by GC-rich codons, an aspect that was not examined in Verma et al.’s study. This can indeed be verified in Fig. 5A, obtained from an analysis of Verma et al.’s large dataset (see “Materials and methods” section). Although eGFP scores are largely spread (reflecting the stochastic nature of the underlying phenomenon), the distributions corresponding to the two highest eGFP scores (4 to 5) are clearly shifted to higher volume values compared to that of the two lowest eGFP scores (1 to 2) (Fig. 5B). Considering average eGFP scores (Fig. 6), further analysis revealed that whenever a glycine is present in positions 3 to 5, the average eGFP scores have a narrower distribution that almost never reach values higher than 3.5 (Fig. 6B; fraction _≥ 3.5_ = 0.4%), while triplets that include at least one alanine (Fig. 6C; fraction_≥ 3.5_: 4.5%) or one serine (Fig. 6D; fraction_≥ 3.5_: 10.1%) have intermediate distributions that are clearly ordered according to volume (gly < ala < ser), suggesting that an absence of side-chain generates the strongest impediment during these early elongation events. At the other end of the size spectrum, a similar inhibitory effect is observed with tryptophan. When this amino acid is present within positions 3 to 5, average eGFP scores are almost never higher than 3.5 (Fig. 6E), similarly to the situation found with glycine. This effect is not observed with phenylalanine (data not shown), which is slightly smaller, suggesting that tryptophan is likely an upper limit in term of bulkiness that can be handled by the PTC, at least during the early rounds of elongation.

Although the amino acid/codon in position 2 was not experimentally investigated by Verma et al., results from an earlier report suggest that the effects of this position on protein synthesis are similar^26^. In both studies, glycine, alanine, serine and tryptophan were all found to hamper translation. Furthermore, both investigations also show that lysine has an agonistic effect, whether it is present in position 2 (Ref.^26^) or in position 3 to 5 (Ref.^3^), a consistency which shows the reliability of these studies.

Expression data related to tryptophan are coherent with a structural constraint associated with incomplete PTC decompaction. The size of the side-chain of this amino acid is so large that it is literally trapped inside the PTC upon compaction (Fig. 3B). Incomplete decompaction is, therefore, expected to hamper the translocation of the terminal CCA from the A side to the P side of the PTC, an effect that could also occur to a lesser extent with other amino acids.

Why then would glycine and amino acids with tiny side-chains also impair subsequent elongation events (Fig. 6B–D)? Because small side chains confer a high flexibility to peptides^27^, these amino acids allow growing peptides to follow suboptimal trajectories, which may prevent them from reaching the exit tunnel, and thus create a jam, or let them exit the PTC through the P site. This may impair the next rounds of elongation, or even lead to peptidyl-tRNA drop-off. Larger side-chains reduce the conformational freedom of peptides, which may help funnel them towards the exit tunnel. This issue would be resolved once the peptide is sufficiently long to be anchored in the tunnel.

Because serine is encoded by two distinct codon families (AGY and TCN, where Y = C or U and N = A, G, C or U), this amino acid provides the opportunity to highlight a possible effect not related to the amino acid itself. It turns out that average eGFP scores for triplets including 1 or more serine (Fig. 6D) are spread to slightly higher values when this amino acid is encoded by AGY codons (fraction_≥ 3.5_: 11.0%), as compared to when it is encoded by TCN codons (fraction_≥ 3.5_: 6.2%), although average eGFP scores are similar (3.03 vs 2.98). This result shows that codons or tRNAs also determine the outcome of early elongation events. A plausible cause for this effect is the sequestration of the used Shine-Dalgarno (SD) sequence (AAGAAG or AAGGAG) by early codons through the formation of a stem-loop (AGY may not base pair with the SD sequence whereas TCN can)^28^. Stenström et al.^26^ pointed out that A-starting codons in second position are associated with a higher efficiency of translation, although the origin of this correlation is unclear.

The above analysis shows that the largest (tryptophan) and the smallest (glycine) amino acids prevent the corresponding peptides from getting high average eGFP scores when they occur in position 3 to 5. Strikingly, the tripeptide with the lowest average score (1.78) is PWG (Fig. 6A), which comprises these two problematic amino acids (tryptophan and glycine), while proline is already known to impair elongation in certain contexts^29–32^. In particular, it was found that the met-pro-tRNA dipeptide configuration on the P site significantly impairs peptide bond formation with puromycin, although no impairment was observed with phe-tRNA as A-site substrate^32^. The above data suggest that a proline in third position could be problematic with full-length A-site substrates in that case. The PWG tripeptide also demonstrates that it is not the overall volume that is critical (which could be incorrectly inferred from the distributions of Fig. 5B), but instead the volume of each amino acid. Global properties also play a significant role. Thus, while the presence of a single glycine in the N_3_–N_5_ triplet is sufficient to prevent any of the corresponding peptide from getting high eGFP scores, the G_3_G_4_G_5_ triplet average score is far from being the lowest: at ~ 2.9, it is slightly above the average of the distribution of Fig. 6B.

**Figure 7:**
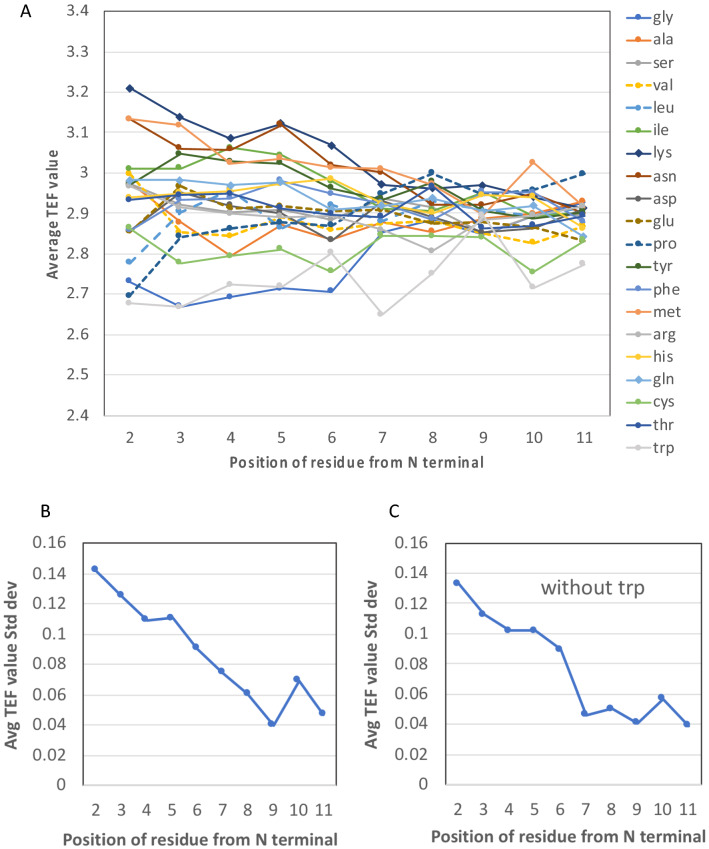
Positional effect of the amino acids on translational efficiency, from position 2 to 11. (**A**) Positional effect on translation of the 20 amino acids. Each TEF average value (see text for a definition of TEF) was established from about 200 to 1500 TEF data, depending on the position and the amino acid. Sequences for which a given amino was present in other position(s) than the one under investigation were removed. (**B**) Positional standard deviation (Std dev) of the average TEF values reported in (**A**). (**C**) Positional standard deviation (Std dev) of the average TEF values reported in A while excluding tryptophan (trp). Plots established from the TEF values listed in Supplementary File 1 of the work published by Sergiev et al.^5^.

In order to get a broader view of the effects of the amino acids on early elongation events, and seek to determine when these early effects cease, we examined a set of data recently published by Osterman et al.^5^. These authors investigated codons 2 to 11, thus providing a larger window of investigation than Verma et al., at the expense of statistics (32,376 fluorescence measurements were achieved, constituting only a tiny fraction of all theoretical sequences). The methodological approach by Osterman et al. is very similar to that of Verma et al. Briefly, a single insert encoding a fluorescent protein preceded by a sequence where pos. 2 to 11 were randomized was expressed in bacteria, which generated a certain level of fluorescence depending on the amount of peptide produced. These bacteria were subsequently sorted into 5 different bins according to the level of fluorescence (defining a variable called ‘TEF’: translation efficiency fraction, which is about equivalent to eGFP scores), and subsequently sequenced to determine the identity of the codons/amino acids in pos. 2–11. Although Osterman et al. already highlighted positional effects associated with each amino acid (their Suppl Fig. S4A), no global quantification was established which could highlight how these effects tend to disappear during the first elongation cycles. Figure 7 show the result of such analysis based on the TEF data by Osterman et al.^5^.

We established the average TEF value at a given position (2 to 11) for each amino acid. Figure 7A reveals that average TEF values for all 20 amino acids are rather spread at position 2, reflecting the various influence that the amino acids can have on the efficiency of translation at this early stage, depending on their physico-chemical properties. These effects tend to dampen as peptides elongate, as revealed by the decrease of the TEF standard deviation (std dev) (Fig. 7B), that reaches a minimal value around position 7. Interestingly a more abrupt jump in std. dev. value is observed between positions 6 and 7 if tryptophan is excluded (Fig. 7C). This analysis shows that the nature of the amino acid on translation efficiency is clearly less important after residue 6. This observed “normalization” suggests that a different regime of translation is reached from position 7, and it is tempting to conclude that, *on average*, this size corresponds to tunnel-inserted peptides. An examination of the pdb 5JU8 ribosome structure with an arrest ErmBL peptide following path 2 (Fig. 4B) reveals that the 7th amino acid is at the level of U2609, which may thus constitue a key residue ensuring the integration of the nascent peptide inside the exit tunnel.

## Discussion

Several studies have highlighted that early rounds of protein elongation are significantly more critical than the subsequent ones for protein synthesis^3,5,23–26^. The present analysis of the induced-fit mechanism of the PTC points out that the conformational freedom of the nascent peptide impairs PTC decompaction during peptide bond formation, which may explain some of these effects. Once anchored in the exit tunnel, a straightened peptide would allow efficient decompaction and thus a faster translocation of the peptidyl-tRNA from the A site to the P site of the PTC. Furthermore, our analysis of protein expression based on eGFP score data by Verma et al.^3^ suggests that small amino acids in positions 3–5 confer a high conformational freedom to nascent peptides that may prevent them from being efficiently funnelled towards the exit tunnel. Additional insights into early elongation events identified by Han et al.^23^ go along this line. While analysing ribosome profiling in mammalian cells, these authors discovered a significant pause in translation at the 5th amino acid residue. They suggested that post-initiation pausing of ribosomes would allow the very first few amino acids to find the correct exit route. Studies on arrest peptides have demonstrated that nascent peptides of that size can already engage significant interactions with the PTC nearby the tunnel’s entrance, where some ribosome residues (such as A2062) play significant role in controlling stalling^33,34^. Han et al.’s findings, indicate that most peptides of 5 residues would reach a critical region of the PTC cavity, which may result in peptide jamming if the N terminal residue is not oriented towards the tunnel’s entrance, a search process that may take some time. This temporary jam could prevent a normal translocation of the A site tRNA 3ʹ end, resulting in a pause. This result appears consistent with the data from Osterman et al., from which we established that a normalization of the TEF signals occurs at position 7. This, we believe, corresponds to the average size from which a nascent peptide gets inserted in the tunnel, and thus has overcome the barrier of the search of the exit tunnel.

Other studies on protein elongation further suggest that early elongation events are necessarily slower than subsequent ones. Recent works by O’Brian et al. have highlighted that the protein segment coming out of the exit tunnel generates a pulling force that is transmitted back to the PTC, where it is thought to stimulated peptide bond formation by lowering the activation energy of the reaction^35,36^. Our analysis suggests that this identified force is also likely to enhance decompaction by straightening the peptide (Fig. 2A), and also to directly promote translocation of the peptidyl-tRNA from the A to the P site of the PTC. Peptide pulling is, furthermore, known to resolve translational stalling that occurs with certain regulatory peptides^37–39^. The mechanism by which translation is arrested with regulatory peptides stems from the interaction of a particular sequence with the exit tunnel^11,39–41^, and can involve additional molecules such as antibiotics^34,42,43^. These interactions preclude the normal progression of the peptide through the tunnel, which perturbs the PTC in such a way that a proper juxtaposition of the aminoacyl-tRNA and the peptidyl-tRNA is impossible, thus preventing peptide bond formation from happening^11,39,44,45^.

Summing up, an important interplay of physical forces is acting at every step of elongation within the PTC and the exit tunnel, these forces being modulated by the sequence of the nascent peptide and the action of antibiotics. When a nascent peptide is not yet anchored in the exit tunnel, no substantial pulling force can help the C terminal amino acyl to get out of a partially decompacted PTC, orient the peptide towards the tunnel and also contribute to moving the tRNA 3ʹ end from the A site to the P site. These constraints are likely the most important reasons why early elongation cycles are slow. The above analysis suggests that three different regimes of translation occur:

1. Before the peptide reaches the exit tunnel. This least efficient regime would prevail until about residue no 5.
2. After the peptide gets inserted in the tunnel. The analysis outlined in Fig. 7 suggests that this regime starts from residue 7 on average. Because peptidyl-tRNA drop-off is expected to be much reduced at that stage due to tunnel insertion, the ‘efficiency’ of translation should be higher from that point.
3. After it emerges from the tunnel. According to O’Brien et al., a pulling force resulting from the interaction of the protein with the solvent and protein folding may enhance peptide bond formation, while our analysis suggests that this force could further straighten the peptide and thus facilitate decompaction and the translocation of the 3ʹ end.

In view of the effect of the translational ramp on the yield of protein synthesis^3^, it is worth noting that no cofactor is known to fix this impediment, similarly as EF-P does it with polyproline and other arresting tracks during elongation^30,31,46,47^, or EF-G, which drives translocation at the level of the decoding center^16,48–51^. Our analysis still suggests a possible way of enhancing the first rounds of elongation: if the initiator methionine is somehow anchored in the exit tunnel, this would enable a nascent peptide to be straightened from the start. This could be achieved by attaching a peptide longer than 4–5 residues to the initiator methionine, or by using an initiator amino acid with a very long side chain. The second possibility could be achieved by taking advantage of the unnatural amino acid technology^52–54^ while using an engineered methionine synthetase specifically modified to recognize azidonorleucine^55,56^. This reactive amino acid could be subsequently modified with a long chain such as polyethylene glycol, that would allow it to anchor in the tunnel.

The fact that no solution to the issue of the translational ramp has emerged throughout evolution suggests that a slow start of elongation is beneficial. As emphasized in several studies^3,23,57^, it may help reduce detrimental ribosome collision during later steps of elongation, these events being caused by ribosome pausing. In an in vitro translation system, where sequences and buffer composition can be optimized, initiator engineering could result in improvements in yields of expressed proteins.

The origin of the translational ramp, whether it is an effect related to the codons, the amino acids, or both, is still debated^3^. The present analysis shows the evidence that at least some of these effects are related to the nascent peptide. Considering that a start codon is positioned along a continuous RNA strand, it seems indeed more likely, from a physical point of view, that penalizing effects at that stage would mostly originate from the nascent peptide. The only clear asymmetry at the level of RNA is the Shine–Dalgarno sequence. This stretch of nucleotides was hypothesized to hamper early elongation events because it binds to the ribosome^58^, an effect that has been recently verified experimentally^59^. Also, DS sequestration by early codons through stem-loop formation may occur^28^.

In conclusion, the present analysis points out that, like a beating heart, the PTC of the ribosome undergoes cycles of compaction–decompaction associated with its induced-fit mechanism. Compaction is required to best align reactive species, while decompaction is necessary to let elongated peptidyl-tRNAs move to the P site of the PTC. Ribosome structures show that decompaction is impaired when the peptide is not yet anchored in the exit tunnel. Combined with an absence of pulling force that would guide the nascent peptide towards the entrance of the tunnel, it may contribute to explain why early rounds of elongation are inefficient.

## Materials and methods

### Analysis of ribosome structures

Crystal and cryo-EM structures of ribosomes complexed with tRNAs and/or minimal substrates analogs were retrieved from the *protein databank* website (https://www.rcsb.org). The *pdb* database was manually screened for all ribosome structures displaying a peptidyl tRNA bound to the A site (A/A and A/P states) and the P site. Representative structures highlighted in the present analysis are pdb 5kcr, 4v5d, 1kqs, 1vy5, and 6wde. Superposition of uninduced (pdb 5kcr) and induced (pdb 4v5d) PTC structures (Fig. 1A) was achieved with the *align* tool of *Pymol* solely based on residues C2452 and A2451 of the PTC.

### Analysis of eGFP expression data

Experimental data highlighting the effect of N_3_-N_5_ residues on the level of expression of eGFP reporters expressed in *E. coli* cells, quantified by the level of fluorescence, were retrieved from Supplementary data 3 published by Djuranovic and collaborators^3^. In this study, *E.coli* cells were sorted based on granularity (SSC-A) and eGFP fluorescence (FITC-A) channels, and split into 5 bins according to the level of fluorescence (med. rel. fluorescence: 20, 120, 600, 3600, 12,000). A total of 215,414 *eGFP* scores, established for 20^3^ = 8000 peptides comprizing a eGFP reported, differing only in positions 3 to 5 (N_3_N_4_N_5_ triplets), were investigated in the present analysis. Average *eGFP* scores (Fig. 6) were established for each particular triplet only if at least 5 independent values were listed. We have investigated the Van der Waals volume^60^ of N_3_–N_5_ triplets as a function of the expression of mRNAs bearing these triplets. See Ref.^3^ for details about experimental procedures.

### Analysis of TEF expression data

Experimental data highlighting the effect of N_2_-N_11_ residues on the level of expression of reporters expressed in *E. coli* cells, quantified by the level of fluorescence, were retrieved from Supplementary Data 1 published by Sergiev et al.^5^.
